# The impact of the Covid-19 pandemic on adult diagnostic neuroradiology in Europe

**DOI:** 10.1007/s00234-021-02722-x

**Published:** 2021-05-11

**Authors:** Marion Smits, M. W. Vernooij, N. Bargalló, A. Ramos, T. A. Yousry

**Affiliations:** 1 Department of Radiology & Nuclear Medicine (Ne-515), Erasmus MC, University Medical Centre Rotterdam, PO Box 2040, 3000 CA Rotterdam, the Netherlands; 2 Department of Epidemiology, Erasmus MC, University Medical Centre Rotterdam, Rotterdam, the Netherlands; 3 Magnetic Resonance Image Core Facility, IDIBAPS and Centre of Diagnostic Imaging (CDIC), Hospital Clinic, Barcelona, Spain; 4 Department of Radiology, Hospital Universitario 12 de Octubre, Madrid, Spain; 5 Division of Neuroradiology and Neurophysics, Lysholm Department of Neuroradiology, UCL IoN, UCLH, London, UK

**Keywords:** COVID-19, Pandemics, Surveys and questionnaires, Personal protection equipment

## Abstract

**Purpose:**

The purpose of this survey was to understand the impact the Covid-19 pandemic has or has had on the work, training, and wellbeing of professionals in the field of diagnostic neuroradiology.

**Methods:**

A survey was emailed to all ESNR members and associates as well as distributed via professional social media channels. The survey was held in the summer of 2020 when the first wave had subsided in most of Europe, while the second wave was not yet widespread. The questionnaire featured a total of 46 questions on general demographics, the various phases of the healthcare crisis, and the numbers of Covid-19 patients.

**Results:**

One hundred sixty-seven responses were received from 48 countries mostly from neuroradiologists (72%). Most commonly taken measures during the crisis phase were reduction of outpatient exams (87%), reduction of number of staff present in the department (83%), reporting from home (62%), and shift work (54%). In the exit phase, these measures were less frequently applied, but reporting from home was still frequent (33%). However, only 22% had access to a fully equipped work station at home. While 81% felt safe at work during the crisis, fewer than 50% had sufficient personal protection equipment for the duration of the entire crisis. Mental wellbeing is an area of concern, with 61% feeling (much) worse than usual. Many followed online courses/congresses and considered these a viable alternative for the future.

**Conclusion:**

The Covid-19 pandemic substantially affected the professional life as well as personal wellbeing of neuroradiologists.

**Supplementary Information:**

The online version contains supplementary material available at 10.1007/s00234-021-02722-x.

## Introduction

The Covid-19 pandemic has a profound worldwide effect on all aspects of human society. Healthcare systems have come under immense pressure with large demands being placed on healthcare professionals. These demands ranged from short terms restructuring of work routines, increasing working hours to being deployed to areas of acute clinical need outside their primary field of practice. Added to these demands are concerns about personal safety at work due to the high infection rate and the potential lack of personal protection equipment (PPE), but also the concern of being potentially the source of infection of loved ones. There is furthermore the psychological impact of the high stress level for those whose work has increased and/or who are directly confronted with the suffering of Covid-19 patients and their families, as well as the impact of the effects of physical and social distancing including social deprivation. Although such demands and concerns are most probably prevalent in all affected countries, they are likely to vary across healthcare fields and countries.

The European Society of Neuroradiology (ESNR) has at its core responsibility to promote the practice, education, and research by neuroradiologists within as well as outside Europe. The ESNR undertook a wide range of activities to support and guide their membership throughout this healthcare crisis, including weekly educational webinars, online courses, and a live online congress. It is however not known to what extent professionals in the field of diagnostic neuroradiology throughout Europe have been affected by the Covid-19 pandemic both in their professional life and their personal wellbeing.

The purpose of this survey was to understand the impact the pandemic has or has had on the work, training, and wellbeing of professionals in the field of diagnostic neuroradiology. These insights may be used to prepare or improve strategies for similar situations and address potential needs and worries that arose from the crisis.

## Methods

An online questionnaire was designed using Survey Monkey (SurveyMonkey Inc., San Mateo, CA, USA). The questionnaire (Table 1 and Supplement 1) featured a total of 46 questions on general demographics (6 questions), the healthcare crisis in its various phases (27 questions) and the situation they were currently in (9 questions), and the numbers of Covid-19 patients at their institution (3 questions). Survey items consisted of single or multiple choice questions, ranking, and provided the option of free text. In addition, respondents were asked to provide the name of their institution. This information was used to assess whether results could have been biased by multiple responses coming from a single institution; all responses were however included in the results.

**Table 1:** Survey questions (see supplement 1 for full survey)

Demographics and phase of pandemic	
Age (162, 97%)	
Gender (165, 99%)	
Country (166, 99%)	
Position (165, 99%)	
Type of institution (166, 99%)	
At what phase of the pandemic are you currently? (165, 99%)	
Institutional Covid-19 patient load	
Approximately how many Covid-19 patients visited your hospital in total up until now? (115, 69%)	
Approximately how many normal ward beds were used at the same time for Covid-19 patients in your hospital at the peak of the crisis (non-cumulative)? (116, 69%)	
Approximately how many intensive care beds were used at the same time for Covid-19 patients in your hospital at the peak of the crisis (non-cumulative)? (117, 70%)	
Measures, safety, and protection	
Crisis phase	Exit phase
What measures are/were introduced during the crisis phase? (146, 87%)	What measures from the crisis phase were/are still in place in the exit phase? (94, 56%)
What specific safety measures are/were established? (146, 87%)	What specific safety measures from the crisis phase were/are still in place in the exit phase? (95, 57%)
Do/did you have sufficient access to personal protection equipment for your work? (146, 87%)	Do/did you have sufficient access to personal protection equipment for your work during the exit phase? (95, 57%)
Do/did you feel safe at work? (146, 87%)	
Change in working conditions	
Crisis phase	Current situation
Are/were you asked to work from home during the crisis phase? (144, 86%)	Do you now work from home more than before the crisis? (92, 55%)
What (if at all) facilities are/were available to work from home during the crisis phase? (146, 87%)	Do you now have more facilities to work remotely than before the Covid-19 crisis (e.g. from home, from different parts of the institution)? (92, 55%)
Do/did you report examinations that were not your speciality, e.g. chest X-ray, chest CT, chest CTA? (145, 87%)	
Do/did you feel comfortable reporting such examinations outside your field of expertise? (145, 87%)	
Does/did your institution ask you to visit or talk to Covid-19 patients or their families? (146, 87%)	
Impact on patient care	
Crisis phase	Exit phase
Are/were the clinical sessions (e.g. oncology meetings) affected in the crisis phase? (146, 87%)	How are/were clinical sessions (e.g. oncology meetings) organized during the exit phase? (95, 57%)
How does/did your institution deal with non-urgent patients in the crisis phase? (146, 87%)	How is/was non-urgent care resumed during the exit phase? (95, 57%)
Is/was there imaging equipment (X-ray, ultrasound, CT or MR scanners) dedicated to only image Covid-19 patients in the crisis phase? (146, 87%)	Is/was there imaging equipment (X-ray, ultrasound, CT or MR scanners) dedicated to only image Covid-19 patients in the exit phase? (95, 57%)
Approximately how much is/was the imaging volume reduced at your institution during the crisis phase? (146, 87%)	Did you make any changes to your imaging protocols (e.g. shorten protocols to handle the backlog or accommodate cleaning of the rooms)? (95, 57%)
Do/did patients stay away at their own initiative (e.g. patients cancelled their appointment themselves, no-show)? (146, 87%)	Do/did patients get tested and/or quarantined for Covid-19 before being admitted to the hospital (e.g. prior to elective surgery/procedures)? (95, 57%)
	When you think about your institution/facility returning to routine operations as Covid-19 restrictions are/were lifted, what are/were your top concerns? (94, 56%)
Is the imaging volume now - back to - normal, i.e. the same as before the crisis? (92, 55%)	
Do you feel that you are prepared for a possible second/further wave? (92, 55%)	
Congresses and education	
Do you follow any online teaching/training/congresses? (92, 55%)	
Do you consider following more courses/congresses online in the future, even if physical courses/congresses are possible again? (92, 55%)	
Impact for residents and fellows	
Crisis phase	Exit phase
If you are a resident or fellow: does/did the crisis phase affect your training? (136, 81%)	If you are a resident/fellow: is/was your training programme - back to - normal during the exit phase? (91, 55%)
	If you are a resident/fellow: how is/was teaching and supervision organized during the exit phase? (90, 54%)
Wellbeing and concerns	
Crisis phase	Current situation
How does/did the crisis phase affect your mental wellbeing? (145, 87%)	How are you feeling now? (92, 55%)
What have been your most important concerns over the past three months? (92, 55%)	
Does your institution provide any (after) care (e.g. mental support programmes, letter from the management to ask how you are)? (92, 55%)	

Numbers in brackets indicate the number of answers received for each question and percentage out of total respondents (*N* = 167)

Due to the variation in Europe, it was expected that respondents would be at different phases of the pandemic, and they were instructed to skip questions on phases that they had not experienced. The early phase was defined as preparing for a potential surge of Covid-19 patients. The acute crisis phase was defined as managing increasing or peak numbers of Covid-19 patients at their institution. The post-acute crisis phase was defined as managing stable but still large numbers of Covid-19 patients at their institution. The exit phase was defined as number of Covid-19 patients in hospital decreasing, starting to resume routine, non-urgent, and/or non-Covid care. Finally, the post-exit phase was defined as the situation where normal care had resumed.

Survey invitations were emailed to all ESNR regular and institutional members (*N* = 5394) and non-members who had expressed their interest in ESNR-activities in the past (*N* = 4134), as well as distributed via professional social media channels (Twitter @ESNRad, 2500 followers; LinkedIn European Society of Neuroradiology, 750 followers) and promoted during the ESNR weekly webinars (approximately 1000 attendees). The survey was held in the summer of 2020 when the first wave had subsided in most of Europe, while the second wave was not widespread; the survey was open for 4 weeks, from 25 July until 21 August 2020.

## Results

### Demographics and phase of pandemic

One hundred and sixty-seven responses were received. Forty-three per cent of respondents disclosed their institution (*N* = 72). From 4 institutions, 2 responses were received, and from 1 institution, 3 responses were received.

Average age of respondents was 45 years (range, 27–74 years; standard deviation [SD], 11 years); 57% (*N* = 94) were female. Most respondents were neuroradiologists (*N* = 119, 72%), followed by general radiologists (*N* = 26, 16%), and residents (*N* = 15, 9.1%). Responses were received from 48 countries (Table 2), most from Italy (*N* = 20, 12%), Spain (*N* = 16, 9.6%), and the Netherlands (*N* = 15, 9.0%). Notably, 45 (27%) responses were from 23 countries outside Europe. Almost two-thirds of respondents worked in a primarily academic institution (*N* = 99, 60%).

**Table 2:** Respondents’ countries of residence (*N* = 166)

Europe	(*N* = 121, 73%)		Outside Europe	(*N* = 45, 27%)	
Italy	20	(12%)	Colombia	6	(3.6%)
Spain	16	(10%)	United States of America	5	(3.0%)
The Netherlands	15	(9.0%)	South Africa	4	(2.4%)
United Kingdom	9	(5.4%)	Brazil	3	(1.8%)
Germany	8	(4.8%)	Argentina	2	(1.2%)
Sweden	6	(3.6%)	Chile	2	(1.2%)
Denmark	5	(3.0%)	Malaysia	2	(1.2%)
Norway	5	(3.0%)	Peru	2	(1.2%)
Portugal	5	(3.0%)	Philippines	2	(1.2%)
Switzerland	5	(3.0%)	Qatar	2	(1.2%)
Serbia	4	(2.4%)	Thailand	2	(1.2%)
France	3	(1.8%)	Australia	1	(0.6%)
Greece	3	(1.8%)	Bhutan	1	(0.6%)
Turkey	3	(1.8%)	Egypt	1	(0.6%)
Austria	2	(1.2%)	India	1	(0.6%)
Belgium	2	(1.2%)	Israel	1	(0.6%)
Bulgaria	2	(1.2%)	Mexico	1	(0.6%)
Russia	2	(1.2%)	New Zealand	1	(0.6%)
Croatia	1	(0.6%)	Pakistan	1	(0.6%)
Finland	1	(0.6%)	Saudi Arabia	1	(0.6%)
Hungary	1	(0.6%)	Ukraine	1	(0.6%)
Ireland	1	(0.6%)	United Arab Emirates	1	(0.6%)
Luxembourg	1	(0.6%)			
Macedonia	1	(0.6%)			
Poland	1	(0.6%)			
Romania	1	(0.6%)			

Most respondents were in the post-exit (*N* = 59, 36%) or exit (*N* = 45, 27%) phase. Fifty respondents were still in the acute (*N* = 27, 16%) or post-acute (*N* = 23, 14%) crisis phase.

### Institutional Covid-19 patient load

**Fig. 1:**
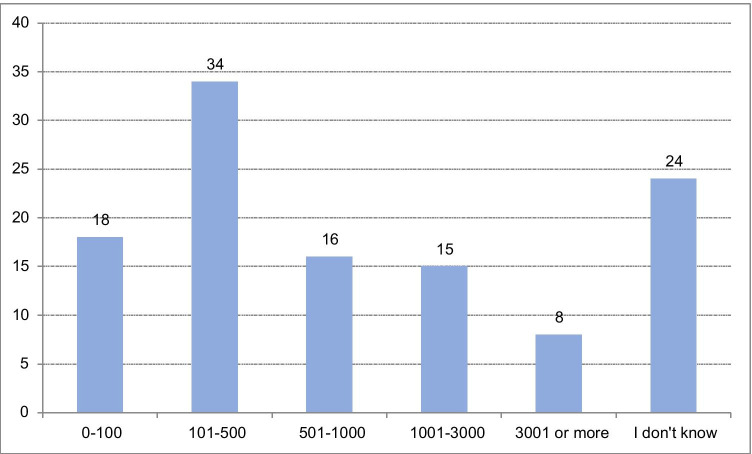
Cumulative number of Covid-19 patients seen in the respondents’ institutions

**Fig. 2:**
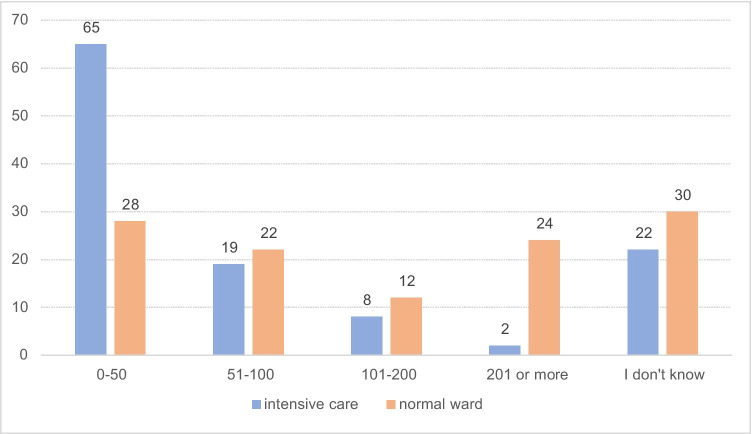
Peak number of beds occupied (non-cumulatively) on the intensive care (blue) and regular ward (orange) by Covid-19 patients in the respondents’ institutions

Most respondents (*N* = 39, 34%) indicated that their institution had seen more than 500 Covid-19 patients cumulatively; almost a similar number (*N* = 34, 30%) had seen 100–500 patients cumulatively (Fig. 1). At the peak of the crisis, the number of beds occupied by Covid-19 patients (non-cumulatively) was up to 100 on regular wards (*N* = 50, 43%) and up to 50 in the intensive care unit (*N* = 65, 56%) in the majority of respondents’ centres (Fig. 2).

### Measures, safety, and protection

Most commonly taken measures during the crisis phase (Table 3) were reduction of outpatient exams (*N* = 128, 87%), reduction of number of staff present in the department (*N* = 122, 83%) and non-essential personnel such as medical students and researchers told to stay away (*N* = 103, 70%), reporting from home (*N* = 91, 62%), and shift work (*N* = 80, 54%). In the exit phase (Table 3), these measures were much less frequently applied, with only reporting from home still being the most frequent measure (*N* = 31, 33%). Other common measures in the exit phase were the re-distribution of workstations throughout the department (*N* = 31, 33%) and reduction of outpatient exams (*N* = 25, 27%), but 22% (*N* = 21) reported that no specific measures were in place anymore (compared with only 1% (*N* = 2)) in the crisis phase.

**Table 3:** General measures taken during the crisis and exit phase (multiple answers possible)

	Crisis phase (*N* = 146)		Exit phase (*N* = 94)	
Reduction of outpatient exams	128	(87%)	25	(27%)
Reduce the number of staff present in the department/hospital	122	(83%)	14	(15%)
Non-essential personnel told to stay away	103	(70%)	18	(19%)
Reporting from home	91	(62%)	31	(33%)
Shift work (working in small teams to reduce cross-infection)	80	(54%)	11	(12%)
Resident training reduced or stopped	62	(42%)	13	(14%)
Workstations distributed/spread out throughout the department	48	(33%)	31	(33%)
Reduction of inpatient exams	47	(32%)	9	(10%)
Deployment to other tasks/departments	45	(31%)	2	(2.1%)
All leave/holiday revoked	40	(27%)	2	(2.1%)
Other	6	(4.1%)	15	(16%)
None, no specific measures were taken	2	(1.4%)	21	(22%)

Safety measures in the crisis phase (Table 4) strongly centred around reducing infection at the workstation, such as not sitting together (*N* = 117, 80%), regular cleaning (*N* = 116, 79%), and providing cleaning products (*N* = 111, 76%). The exposure to potentially infectious persons was limited by screening all patients for symptoms (*N* = 115, 97%) and restricting the access of visitors to the hospital (*N* = 106, 73%) or reporting room (*N* = 72, 49%). These were still the most common safety measures in the exit phase (Table 4).

**Table 4:** Specific safety measures during the crisis and exit phase (multiple answers possible)

	Crisis phase (*N* = 146)		Exit phase (*N* = 95)	
Distancing measures (e.g. not sitting together at the workstation)	117	(80%)	71	(75%)
Regular cleaning if reporting rooms, desks, workstations	116	(80%)	68	(72%)
Screening of patients for Covid-19 symptoms	115	(79%)	67	(71%)
Providing cleaning products	111	(76%)	79	(83%)
No/limited visitors allowed in the hospital	106	(73%)	42	(44%)
Maximum number of people allowed in meeting/reporting room	94	(64%)	54	(57%)
Department restricted to essential staff only	72	(49%)	14	(15%)
Staff separated into separate rooms	64	(44%)	28	(30%)
Other	8	(5.5%)	6	(6.3%)
None, no specific measures were taken	1	0.7%)	3	(3.2%)

Access to PPE (Table 5) was sufficient during the entire crisis phase for fewer than half of the respondents (*N* = 70, 48%), although for a further 40% (*N* = 59), this access improved during the crisis phase. Only 9 respondents (6%) reported having no or deteriorating access to PPE. During the exit phase, the vast majority (*N* = 83, 87%) had sufficient access to PPE (Table 5).

**Table 5:** Sufficient access to personal protection equipment during the crisis and exit phase

	Crisis phase (*N* = 146)		Exit phase (*N* = 95)	
Yes	70	(48%)	83	(87%)
Not at the beginning of the crisis, but this improved	59	(40%)	–	
Only at the beginning of the crisis, but this got worse	3	(2.1%)	–	
No	6	(4.1%)	6	(6.3%)
Not applicable, I do not have any contact with patients	8	(5.5%)	6	(6.3%)

While 81% (*N* = 118) felt safe at work during the crisis phase, a substantial proportion of respondents did not (*N* = 28, 19%), with reasons stated as lack of sufficient PPE (*N* = 12), failure of testing and triaging strategy resulting in unprotected contact with infected persons (*N* = 6), general concerns about getting infected (*N* = 6), and conditions in the workplace not allowing for physical distancing (*N* = 5).

### Change in working conditions

The majority of respondents were asked to work from home during the crisis phase (*N* = 91, 63%), either in part (*N* = 79) or fully (*N* = 12). Tools that were (made) available for working from home at that time were mostly online meeting software (*N* = 71, 49%) and remote access to work computers (*N* = 65, 45%). Fewer than a quarter had a fully equipped home reporting system available (*N* = 34, 23%). Nine respondents (6%) indicated that they had no facilities to work from home. Of those that answered the questions about their current situation (*N* = 92), fewer than half now work more from home more than they did before the crisis (*N* = 39, 42%). Most have now more access to these tools (Table 6) than before the crisis.

**Table 6:** Increase of facilities to work remotely compared to before the Covid-19 crisis (multiple answers possible)

	Total *N* = 92	
Not applicable, do not work from home	22	(24%)
No	30	(33%)
Yes: online meeting software, provided/paid for by the institution	32	(35%)
Yes: remote access to work computers	22	(24%)
Yes: fully equipped home reporting system	11	(12%)

Thirty-five per cent (*N* = 51) respondents indicated they reported examinations outside their speciality during the crisis phase. Most felt comfortable doing so (*N* = 43, 75%).

Twenty respondents (14%) were asked to visit or talk to Covid-19 patients or their families during the crisis phase.

### Impact on patient care

There was a dramatic effect on clinical sessions (e.g. oncology meetings) during the crisis phase; only 5 respondents (3%) indicated no change (Table 7). These sessions were mostly held online (*N* = 58, 38%) and/or with only a limited number of participants allowed to be present on site (*N* = 39, 27%), reduced in number (*N* = 46, 32%), or completely cancelled (*N* = 29, 20%). During the exit phase, only 31% (*N* = 29) of clinical meetings had gone back to a “normal” on site presence, with the introduction of some safety measure such as masks and/or physical distancing (Table 7). In most cases, the meetings were held in a hybrid fashion (*N* = 37, 39%). Only 5% (*N* = 5) indicated that the meetings were still (partially) cancelled.

**Table 7:** Effect on clinical sessions (multiple answers possible)

	Crisis phase (*N* = 146)		Exit phase (*N* = 95)	
All sessions were cancelled	29	(20%)	5	(5.3%)
Some sessions were held, but many were cancelled	46	(32%)	–	
Sessions were held but the number of patients was reduced	30	(21%)	–	
Sessions were held physically, but with a limited number of participants	39	(27%)	25	(26%)
Sessions were held online	56	(38%)	28	(29%)
Sessions were held hybrid (partly online, partly physical)	–		37	(39%)
No impact/back to normal	5	(3.4%)	29*	(31%)
Do not know	8	(5.5%)	1	(1.1%)

*Two respondents indicated that face masks were worn

Non-urgent care was mostly continued for a limited selection of cases during the crisis phase (*N* = 85, 58%), but a substantial amount of non-urgent care was (mostly) cancelled during this phase (*N* = 53, 36%) with only 6 respondents (4%) reporting no change in non-urgent care during the crisis phase. During the exit phase, the restart of care was mostly for selected patients (e.g. oncology) first (*N* = 48, 51%) rather than for all patients at the same time (*N* = 35, 37%).

**Fig. 3:**
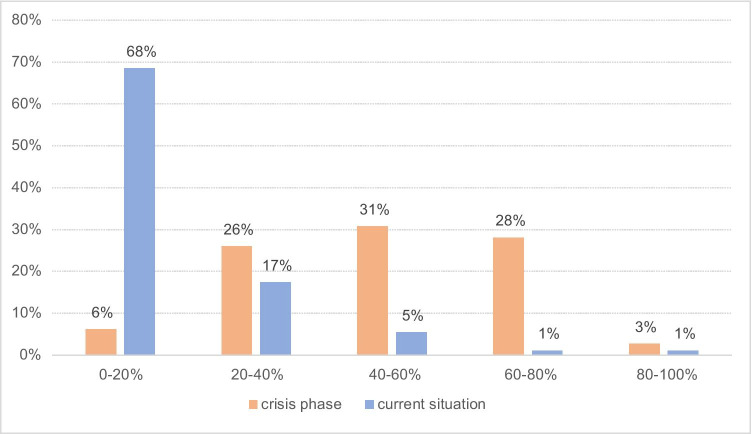
Percentage reduction in imaging examinations during the crisis phase (orange) and at the time of answering the survey (blue). Note that at the time of answering the survey 62% of respondents indicated that imaging volume had returned to normal; these responses are included in the 0–20% bracket

Respondents also reported a substantial reduction in imaging volume during the crisis phase (Fig. 3) by at least 20% in 88% (*N* = 128) and by more than 60% in 31% (*N* = 45). Many respondents (*N* = 90, 62%) indicated that patients stayed away at their own initiative. At the time of answering the survey, 62% (*N* = 57) indicated that their imaging volume was back to normal (Fig. 3). Where the imaging volume was still reduced, this was mostly by about 20–40% (*N* = 16, 50%), while only 6% (*N* = 2) reported a reduction compared to normal of 60%. Some indicated that the reduction could also be ascribed to the summer holiday at that time.

During the crisis phase, just under half of the respondents (*N* = 71, 49%) indicated that imaging equipment was dedicated to only image – suspected – Covid-19 patients. These were primarily CT and X-ray (*N* = 49, 69% and *N* = 40, 56%, respectively) as well as ultrasound (*N* = 19, 27%) and even MRI (*N* = 12, 17%). During the exit phase, this situation had changed, with only 19% (*N* = 18) respondents indicating the dedicated use of imaging equipment for – suspected – Covid-19 patients, although some indicated dedicated time slots for Covid-19 patients on equipment that was otherwise in routine use. Again, dedicated equipment was primarily CT and X-ray (*N* = 10, 56% for both modalities), followed by ultrasound (*N* = 7, 39%).

With the resumption of routine care during the exit phase, most reported no change in their imaging protocols (*N* = 75, 79%). Those that did report a change were more or less equally divided between shortening the protocols to accommodate more patients to be scanned and/or allow cleaning of rooms in-between patients (*N* = 9, 45%) and increasing the duration of time slots or time between examinations to allow for cleaning the room between patients (*N* = 8, 40%). Two reported the implementation of dedicated Covid-19 imaging protocols. In this phase, most reported that patients routinely got tested for Covid-19 and/or quarantined before being admitted to the hospital for elective procedures (*N* = 67, 71%).

The majority of respondents indicated that they felt sufficiently prepared for a further wave (*N* = 78, 85%). Concerns raised by those who did not feel as such included – mental – exhaustion (*N* = 3), uncertainty about the development of the pandemic (*N* = 2), the lack of home reporting stations (*N* = 1), and unsafe working conditions (*N* = 1).

### Congresses and education

A large majority of respondents had participated in online courses and conferences (*N* = 72, 78%). A similarly large proportion (*N* = 69, 75%) indicated that they would consider online courses and conferences as an alternative to physical courses and congresses in the future, even if large physical meetings become possible again. One participant made the specific comment that they learn better in their own time, being able to pause/rewind and look up things as they go along. This person also noted that as a trainee, they cannot always get time off work so it would be really beneficial to attend the courses and meetings in their own time. The benefit for the environment was also noted.

### Impact for residents and fellows

Questions on this topic were answered by 20 respondents (15%); others skipped these questions (*N* = 31) or indicated that they were not a resident/fellow (*N* = 116). Ten respondents indicated no impact of the pandemic on their training in the acute phase, 7 reported a reduction in work/cases, 1 cancellation of external rotations, 1 cancellation of interdepartmental meetings, and 1 delay in obtaining the European Diploma in Neuroradiology. The vast majority of those who answered this question for the exit phase (*N* = 9) indicated that their training programme was back to normal (*N* = 8, 89%). Only 1 person indicated that teaching was still reduced compared to normal. No specific measures were taken during teaching or supervision according to 50% (*N* = 6) of responding residents/fellows; in case measures were taken, these were remote supervision (*N* = 4, 33%) or teaching (*N* = 3, 25%) to ensure physical distancing.

### Wellbeing and concerns

**Fig. 4:**
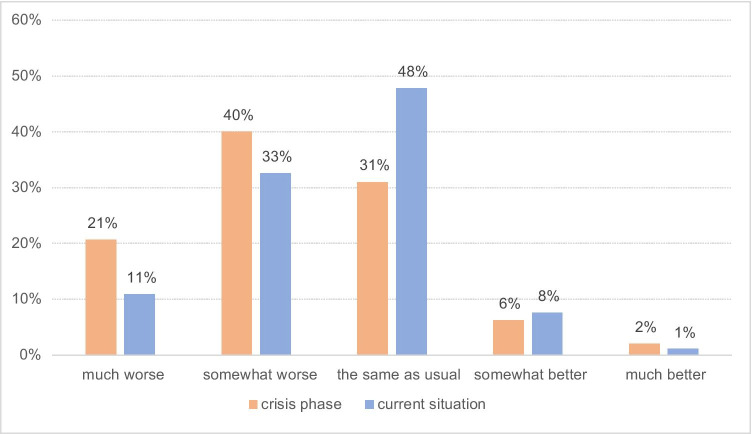
Mental wellbeing as compared to normal during the crisis phase (orange) and at the time of answering the survey (blue)

Asked about their mental wellbeing, 61% of respondents indicated they felt worse or much worse than usual (*N* = 58, 40%, respectively, *N* = 30, 21%) during the crisis phase; 31% (*N* = 45) felt the same as usual (Fig. 4). These numbers were improved at the time of filling out the survey, with 48% (*N* = 44) feeling the same as usual during that time of the year, although 44% still felt worse (*N* = 30, 33%) or much worse (*N* = 10, 11%) compared to usual (Fig. 4).

**Fig. 5:**
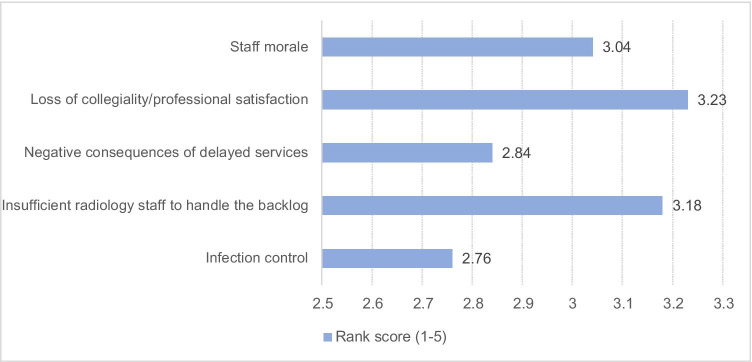
Concerns about returning to routine operations as Covid-19 restrictions are lifted (ranked from 1 = lowest to 5 = highest concern)

Asked about their concerns in the past 3 months (Table 8), the vast majority reported worries about their personal or family health and safety (*N* = 66, 72%). Adapting to Covid-19 operational changes was also a major source of concern (*N* = 46, 50%) as were non-work obligations due to Covid-19 (*N* = 32, 35%), impact on personal finances (*N* = 25, 27%), and on job security (*N* = 218, 20%). Of the 5 presented concerns about returning to routine operations in the exit phase, loss of collegiality/professional satisfaction and insufficient staff to handle the volume of rescheduled cases ranked first and second (Fig. 5).

**Table 8:** Most important concerns over the past 3 months (multiple answers possible)

	Total *N* = 92	
Impact on academic career advancement	10	(11%)
Impact on training	5	(5.4%)
Impact on personal finances	25	(27%)
Job security	18	(20%)
Inability to conduct research/fulfil grant requirements	14	(15%)
Adapting to Covid-19 operational changes	46	(50%)
Personal/family health and safety	66	(72%)
Non-work obligations impacted by Covid-19	32	(35%)
None, no concerns	1	(1.1%)
Other*	8	(8.7%)

*Logistics in the department (*N* = 3), cancellation of international meetings (*N* = 1), meetings/discussions with team (*N* = 1), education of residents (*N* = 1), not being able to do courses/exam (*N* = 1), world health and economics (*N* = 1)

Only 34% of respondents (*N* = 31) indicated that their institution provided some form of care or aftercare, consisting primarily of some form of mental support (*N* = 27, 87%). Other forms of care that were mentioned were information/discussion sessions (*N* = 3), surveys by the institution (*N* = 1), and support on a personal level by their superior (*N* = 1).

## Discussion

Our survey shows that the Covid-19 pandemic, in addition to its impact on patient care, substantially affected the professional life as well as personal wellbeing of neuroradiologists throughout Europe and beyond, many of which had lasting effects in later phases after the first wave. There was a dramatic reduction in imaging examinations, a rapid transition to alternative working patterns, and the implementation of numerous measures to reduce the risk of infections both for patients and healthcare professionals. While we found that many respondents seemed to have adapted well to these changes, many also seemed to have suffered mentally with concerns regarding various aspects of their work and the workplace, as well as the health and safety of themselves and their loved ones. Not only did clinical work transition to a remote or online format, so did education and congresses, and this was mostly received positively.

### Context, strengths, and weaknesses of the survey

This survey was primarily set out to assess the impact of the Covid-19 pandemic on work, training, and wellbeing of professionals in the field of diagnostic neuroradiology. Responses were mostly received from neuroradiologists at (academic) institutions with substantial Covid-19 case load and from a wide geographical distribution, from within but also outside Europe. Responses did not seem to be biased by experiences from a particular institution, as only from 4 centres 2, and from 1 centre, 3 responses were received. However, it should be noted that with a response rate of only 1.5%, findings are not generalisable and should be interpreted with care.

At the time of the survey, most respondents had – recently – left the first Covid-19 wave behind, but the second wave had not yet started in most of Europe. This setting provides a good context for surveying the initial strategies employed when this healthcare crisis hit institutions more or less unexpectedly.

### Impact on the healthcare system, the workplace, and pattern of work

That the pandemic dramatically impacted the healthcare system throughout most of the world is beyond any doubt. Numerous studies and healthcare directives have indicated how routine non-urgent and even urgent care has been and still is substantially scaled down [2–5]. The true extent of the long-term damage on global health remains to be seen, but delayed diagnoses and treatments as well as the impact of lockdowns on mental health and lifestyle are expected to substantially reduce quality of life in many years to come. Our survey adds to published findings, with a reported 20–60% reduction of imaging procedures during the crisis phase which had only partially recovered in the exit phase in the summer of 2020 [10]. Note that while this survey was sent to the European neuroradiological community, questions regarding imaging procedures encompassed *all* imaging procedures at their institution, and survey results are thus not restricted to neuroimaging procedures only. Imaging is a crucial component of healthcare, providing guidance on diagnosis and treatment both in the initial stage of disease and during surveillance during or after treatment. The reduction of imaging procedures can thus be seen as a proxy for the proportion of missed or delayed diagnoses and suboptimal patient management.

In the workplace, a multitude of safety measures were employed, primarily aimed at improving hygiene and reducing person to person contacts, such as reducing the number of staff present in the department, working in shifts, working from home, and banning non-essential persons from the workplace. While only a small proportion of respondents seemed sufficiently equipped to fully work from home, many were provided with tools to allow some form of remote working. The transition to remote work was not restricted to reporting from home or online meetings but also included the online or hybrid continuation of clinical sessions such as tumour board meetings. This indicates that overall, there has been a rapid adaptation to a new pattern of neuroradiological work that was partly maintained after the crisis phase to ensure a safe and efficient working environment throughout the pandemic.

While the crisis clearly had a profound effect on the entire healthcare system, 85% felt they were sufficiently prepared for a further wave of infections indicating that new ways of working were adequately implemented within a short period of time. Neuroradiologists were sometimes involved in reporting examinations outside their specialty, presumably to aid more heavily affected subspecialties such as chest radiology, and most were comfortable in doing so. This speaks to a great flexibility and resilience of the profession in the face of a healthcare crisis.

### Impact on the neuroradiologist: Safety and wellbeing

Despite known shortages of PPE throughout the world, most of our respondents reported to have had sufficient access to PPE, although not always from the start of the crisis, and the majority felt safe at work. It should however be noted that direct contact with patients amongst diagnostic neuroradiologists is generally limited, thus reducing the need for PPE, especially in comparison with radiographers and other frontline workers in other fields of radiology or other clinical specialties. In light of this, it is striking that almost 20% of respondents did not feel safe at work.

An area of real concern is the – mental – wellbeing of healthcare professionals. While it seems that safety in the workplace was generally well guarded, many respondents experienced a clear reduction in their – mental – wellbeing, with almost two-thirds feeling worse to much worse during the crisis compared to usual. Although this number had improved at the time of filling out the survey, just under half still felt worse or even much worse. Respondents were mostly worried about their personal and family’s health and safety, as well about adapting to the changes at work, non-work obligations due to Covid-19, personal financial worries, and concerns about job security. This is in line with findings from a survey amongst ICU providers in the USA, which found worries about transmitting COVID-19 to family/community in 66%, emotional distress/burnout in 58%, and insufficient PPE in 40% [11].

The effect of the pandemic in general is closely intertwined with specific professional issues arising from the healthcare crisis. In a survey amongst almost 2000 employees of two Finnish medical institutions, almost half were found to have a variety of work-related stress and anxiety issues that were higher than the general population’s and were also independent of whether employees had direct patient contact or not [9].

### Training, education, congresses

The impact on training and education of residents and fellows cannot be assessed with certainty, as only 15% of respondents fell in this category. Half of these reported no impact of the pandemic on their training, while the other half were primarily affected by a reduction in available work and cases during the crisis phase. Overall, respondents (both radiologists and residents) adapted well to the online format into which many courses and congresses were transformed, many having attended online education and congresses and also intending to continue to do so even when in-person meetings are possible again in the future.

Online activities, congresses, and education were very well received. Other studies had similar findings. In a survey held after a highly successful online course on liver imaging, the vast majority (97%) of 487 respondents from 37 countries found the online course beneficial, and 84% said that they would attend future virtual conferences even if in-person conferences resume [1]. In a survey amongst 891 neurosurgeons from 96 countries, most perceived virtual learning as positive, and most also indicated that virtual learning would likely replace on-site events, at least partially [6]. Geographically, those from lower-income countries as well as those from parts of Europe and Central Asia supported to this transition towards and further development of a virtual teaching environment. Also, outside the field of medicine, a poll from *Nature* (https://www.nature.com/articles/d41586-020-01489-0), after one of the first meetings to go online due to the pandemic, indicated that over 80% of 485 attendees of the American Physical Society would be willing to attend an online conference in the future.

### What are the lessons learned from the ESNR community’s response to the 6 months of the pandemic?

First, it is concerning that such substantial reductions in imaging procedures were reported, with as yet to determine negative health effects and that generally no adaptations were made to deal with the backlog that arose during the crisis phase. One option applied by some respondents, and potential strategy to consider for possible further waves or similar situations, is to shorten protocols to accommodate larger numbers of imaging volumes.

Second, concerns about safety in the workplace can and should be specifically addressed in future similar situations. Almost 20% of our respondents did not feel safe at work. In the previously mentioned survey amongst ICU providers, the lack of PPE was found to be the strongest predictor of feeling that the hospital is unable to keep providers safe, worries about transmitting infection to families/communities, and significantly associated with emotional distress and burnout [11].

Third, given the impact of the pandemic in general and high demands placed on healthcare professionals in particular, it is striking that only a minority of institutions seemed to provide some form of (after)care. These issues only become more prominent with the prolonged duration of the crisis where – mental – exhaustion will become a real concern [1, 7, 8]. Where institutions fail to provide care programmes, departments could organise these themselves, and failing that, colleagues could actively try to support each other.

Finally, it is clear that online activities are here to stay with mentioned benefits including flexibility, inclusiveness, and reduced cost both financially and to the environment. International societies and communities could and should take such preferences into account by further developing – partially – online formats for their activities. An important aspect to be considered here is the interaction between participants, within but particularly outside sessions. While online meetings lack the benefits of face-to-face networking or spontaneous coffee queue conversations, online interaction is not impossible, and in fact, for many – particularly junior – attendees, approaching a fellow or more senior scientist might be less intimidating online than face to face. Such new avenues of increased inclusiveness could be explored and developed further in the future.

## Conclusion

In conclusion, the European neuroradiological community has been substantially impacted by the Covid-19 pandemic, but has responded with great flexibility and resilience, and seems to take valuable tools such as remote working and online education on board for the future.

## Supplementary Information

ESM 1 (DOCX 22 kb)

## Data Availability

Can be requested from the corresponding author, except for information on the respondent’s institution.
