# Supplementary material for: The impact of the Covid-19 pandemic on adult diagnostic neuroradiology in Europe
Source: Neuroradiology. 2021 May 11;64(1):31–42. doi: 10.1007/s00234-021-02722-x (PMC8110687; doi:10.1007/s00234-021-02722-x)
Supplement: Supplementary file 1 — (DOCX 22 kb) [file 234_2021_2722_MOESM1_ESM.docx]

Supplement 1

**Survey on the impact of the Covid-19 Pandemic on adult diagnostic neuroradiology**

# Introduction

Thank you for taking the time to participate in this survey. All data will be analysed anonymously and confidentially and stored securely. The survey takes ± 15 min to complete. You can skip questions that don’t apply to you, but the more complete you can be the better. Please make sure to submit your answers at the end of the survey.

The results of this survey will help us as a community to understand the impact the pandemic has or has had on our work, our training, and our wellbeing as professionals in the field of diagnostic Neuroradiology. These insights may be used to prepare or improve strategies for similar situations (e.g. further Covid-19 waves) and address potential needs and worries that arose from the crisis.

Take care & stay safe,

Tarek Yousry, Marion Smits, Meike Vernooij, Ana Ramos, Nuria Bargallo
*The ESNR Subcommittee on Diagnostic Neuroradiology*

# A. Demographics & phase of the pandemic

- Age (free text)
- Gender: male/female/I’d rather not say
- Country: drop down menu of all European countries plus other (free text)
- Position (resident, fellow, neuroradiologist, general radiologist, other - free text)
- Institution: Primarily academic/non-academic position

At what phase of the pandemic are you currently?

- Early phase (preparing for the surge of Covid-19 patients)
- Acute crisis phase (managing increasing or peak numbers of Covid-19 patients in hospital/intensive care)
- Post-acute crisis phase (stable but still large number of Covid-19 patients in hospital/intensive care)
- Exit phase (number of Covid-19 patients in hospital decreasing, starting to resume routine/non-urgent/non-Covid care)
- Post-exit phase (normal care has been resumed)
- None of the above: not affected by a surge of Covid-19 patients nor anticipating this in the near future → go to section F

# B. Crisis phase

1. What measures are/were introduced during the crisis phase? (multiple answers possible)

- Reduce the number of staff present in the department/hospital
- Shift work (working in small teams to reduce the risk of cross-infection)
- Workstations distributed/spread out throughout the department
- Reporting from home
- Reduction of outpatient exams
- Reduction of inpatient exams
- Non-essential personnel (e.g. medical students, researchers) told to stay away
- Resident training reduced or stopped
- Deployment to other tasks/departments (e.g. Covid-19 wards or intensive care)
- All leave/holiday revoked
- Other (free text)
- None, no specific measures were taken

2. What specific safety measures are/were established? (multiple answers possible)

- Regular cleaning of reporting rooms, desks, workstations
- Providing cleaning products
- Staff separated into separate rooms
- Distancing measures (e.g. not sitting together at the same workstation)
- Maximum number of people allowed in meeting/reporting room
- Department restricted to essential staff only (e.g. referring clinicians not allowed to enter the department)
- Screening of patients for Covid-19 symptoms upon entering the hospital/department
- No/limited visitors allowed in the hospital
- Other (free text)
- None, no specific measures were taken

3. Do/did you have sufficient access to personal protection equipment for your work?

- Yes, during the entire crisis
- Not at the beginning of the crisis but this improved during the crisis
- Only at the beginning of the crisis, but this got worse during the crisis
- No
- Not applicable, I don’t have any contact with patients

4. Do/did you feel safe at work?

- Yes
- No (please clarify) (free text)

5. How does/did the crisis phase affect your mental wellbeing?

- I feel/felt much worse (e.g. tired, overwhelmed, stressed, anxious, depressed) than usual
- I feel/felt somewhat worse than usual
- I feel/felt the same as usual
- I feel/felt somewhat better than usual
- I feel/felt much better than usual

6. If you are a resident or fellow: Does/did the crisis phase affect your training?

- Yes (please clarify) (free text)
- No
- Not applicable: I’m not a resident/fellow

7. Are/were you asked to work from home during the crisis phase?

- Yes, to work fully from home
- Yes, to work in part from home
- No, not to work from home at all

8. What (if at all) facilities are/were available to work from home during the crisis phase? (multiple answers possible)

- Not applicable, I don’t/didn’t work from home during the crisis
- None
- Online meeting software, provided/paid for by the institution (e.g. Zoom, Microsoft Teams, Skype for Business; not the free versions)
- Remote access to work computers (remote desktop, remote workstation)
- Fully equipped home reporting system
- Other (free text)

9. Do/did you report examinations that were not your speciality, e.g. chest X-ray, chest CT, chest CTA?

- Yes → go to question 10
- No → go to question 11

10. Do/did you feel comfortable reporting such examinations outside your field of expertise?

- Yes
- No
- Not applicable, I didn’t report examinations outside my field of expertise

11. Does/did your institution ask you to visit or talk to Covid-19 patients or their families?

- Yes
- No

12. Are/were the clinical sessions (e.g. oncology meetings) affected in the crisis phase? (multiple answers possible)

- Yes: they were stopped entirely
- Yes: only some clinical sessions were held, but many were cancelled
- Yes: the number of patients was very much reduced
- Yes: only a limited number of participants was allowed to be present
- Yes: they were held online
- Other (free text)
- No
- I don’t know

13. How does/did your institution deal with non-urgent patients in the crisis phase?

- They were mostly or all cancelled
- Only a limited selection of patients was still seen (e.g. oncology)
- There was no change
- Other (free text)
- I don’t know

14. Is/was there imaging equipment (X-ray, ultrasound,CT or MR scanners) dedicated to only image Covid-19 patients in the crisis phase?

- Yes → follow-up question: what equipment (tick boxes or free text)?
- No
- I don’t know

15. Approximately how much is/was the imaging volume reduced at your institution during the crisis phase?

- 0-20% reduction compared to normal
- 20-40% reduction compared to normal
- 40-60% reduction compared to normal
- 60-80% reduction compared to normal
- 80-100% reduction compared to normal
- I don’t know

16. Do/did patients stay away at their own initiative (e.g. patients cancelled their appointment themselves, no-show)?

- Yes → follow-up question: what examinations? (free text)
- No
- I don’t know

17. Have you reached the exit/post-exit phase (i.e. are you starting to resume or have you resumed routine care)?

- Yes → continue to section C
- No → continue to section E

# C. Exit phase: starting to resume normal care under Covid-19 conditions

1. What measures from the crisis phase were/are still in place in the exit phase? (multiple answers possible)

- Reduce the number of staff present in the department/hospital
- Shift work (working in small teams to reduce the risk of cross-infection)
- Workstations distributed/spread out throughout the department
- Reporting from home
- Reduction of outpatient exams
- Reduction of inpatient exams
- Non-essential personnel (e.g. medical students, researchers) told to stay away
- Resident training reduced or stopped
- Deployment to other tasks/departments (e.g. Covid-19 wards or intensive care)
- All leave/holiday revoked
- Other (free text)
- None, no specific measures are in place

2. What specific safety measures from the crisis phase were/are still in place in the exit phase? (multiple answers possible)

- Regular cleaning of reporting rooms, desks, workstations
- Providing cleaning products
- Staff separated into separate rooms
- Distancing measures (e.g. not sitting together at the same workstation)
- Maximum number of people allowed in meeting/reporting room
- Department restricted to essential staff only (e.g. referring clinicians not allowed to enter the department)
- Screening of patients for Covid-19 symptoms upon entering the hospital/department
- No/limited visitors allowed in the hospital
- Other (free text)
- None, no specific measures were taken

3. When you think about your institution/facility returning to routine operations as Covid-19 restrictions are/were lifted, what are/were your top concerns? Please select your top 3

- Infection control/risk of exposure (e.g. how to operationalise physical distancing, sanitation, staggering of patients, waiting rooms, continued use of personal protection equipment)
- Insufficient radiology staff to handle the volume of rescheduled cases in backlog (e.g. due to furloughs, cuts, lack of childcare options)
- Negative consequences to patients of delayed services (e.g. delayed screening, delayed procedures)
- Loss of collegiality, loss of professional satisfaction (e.g. due to decreased in-person consultations and conferences, direct interaction with trainees)
- Staff morale (e.g. the impact of continued fear of exposure, unhappiness about how staff was treated during the Covid-19 crisis, salary cuts, lack of personal protection equipment)
- None of the above, I have/had no concerns.

4. Do/did you have sufficient access to personal protection equipment for your work **during the exit phase**?

- Yes
- No
- Not applicable, I don’t have any contact with patients

5. If you are a resident/fellow: is/was your training programme - back to - normal **during the exit phase**?

- Yes
- No → follow-up question: what is/was - still - different from normal?
- Not applicable: I’m not a resident/fellow

6. If you are a resident/fellow: how is/was teaching and supervision organised **during the exit phase**? (multiple answers possible)

- As before the crisis, no specific measures are in place
- Supervision is/was done remotely, to make sure that physical distancing can be maintained
- Teaching is/was done online, to make sure that physical distancing can be maintained
- Not applicable: I’m not a resident/fellow

7. How are/were clinical meetings (e.g. oncology meeting) organised **during the exit phase**? (multiple answers possible)

- They are/have gone back to normal, as before the Covid-19 crisis
- They are/were held online
- They are/were held physically, but with a limited number of participants
- They are/were hybrid, i.e. with a limited number of physical participants, and others participating online
- They are/were still cancelled
- Other (free text)

8. How is/was non-urgent care resumed during the exit phase?

- All care was/is restarted for all patients at the same time
- Selected care (e.g. oncology) was/is restarted first
- Other (free text)
- I don’t know
- Not applicable, we didn’t stop our non-urgent care during the crisis phase

9. Is/was there imaging equipment (X-ray, ultrasound,CT or MR scanners) dedicated to only image Covid-19 patients **in the exit phase**?

- Yes → follow-up question: what equipment (free text)?
- No
- I don’t know

10. Did you make any changes to your imaging protocols (e.g. shorten protocols to handle the backlog or accommodate cleaning of the rooms)?

- Yes → follow-up question: please clarify (free text)
- No

11. Do/did patients get tested and/or quarantined for Covid-19 before being admitted to the hospital (e.g. prior to elective surgery/procedures)?

- Yes
- No

# D. Current situation

1. What have been your most important concerns over the past three months? (multiple answers possible)

- Impact on academic career advancement
- Impact on my training
- Impact on personal finances
- Job security
- Inability to conduct research or fulfil grant requirements
- Adapting to Covid-19 operational changes
- Personal/family health and safety
- Non-work obligations impacted by Covid-19 (child care, care for - elderly - family members)
- Other: free text
- None, I didn’t have any concerns

2. Do you now work from home more than before the crisis?

- Yes
- No

3. Do you now have more facilities to work remotely than before the Covid-19 crisis (e.g. from home, from different parts of the institution)? (multiple answers possible)

- Not applicable, I don’t work from home
- No, I don’t have any more facilities than before the crisis
- Yes: I now have online meeting software, provided/paid for by the institution (e.g. Zoom, Microsoft Teams, Skype for Business; not the free versions)
- Yes: I now have remote access to work computers (remote desktop, remote workstation)
- Yes: I now have a fully equipped home reporting system
- Yes: other (free text)

4. Is the imaging volume now - back to - normal, i.e. the same as before the crisis?

- Yes
- No → follow-up question: still reduced by ..%
- I don’t know

5. Do you follow any online teaching/training/congresses?

- No
- Yes → follow-up question, please specify (free text)

6. Do you consider following more courses/congresses online in the future, even if physical courses/congresses are possible again?

- No, I would primarily go to physical courses/congresses if these are possible again
- Yes, I would consider these as an alternative to physical courses/congresses
- Other (free text)

7. How are you feeling now?

- Much worse (e.g. tired, overwhelmed, stressed, anxious, depressed) than I usually feel at this time of the year
- Somewhat worse than I usually feel at this time of the year
- The same as I usually feel at this time of the year
- Somewhat better than I usually feel at this time of the year
- Much better than I usually feel at this time of the year

8. Does your institution provide any (after)care (e.g. mental support programmes, letter from the management to ask how you are)?

- No
- Yes → follow-up question: please specify (free text)

9. Do you feel that you are prepared for a possible second/further wave?

- Yes
- No (please specify) (free text)

# E. Numbers of Covid-19 patients in your institution

1. Approximately how many Covid-19 patients visited your hospital in total up until now?

- 0-100
- 100-500
- 500-1,000
- 1,000-3,000
- 3,000 or more
- I don’t know

2. Approximately how many **normal ward beds** were used at the same time for Covid-19 patients in your hospital at the peak of the crisis (non-cumulative)?

- 0-50
- 50-100
- 100-200
- 200 or more
- Don’t know

3. Approximately how many **intensive care beds** were used at the same time for Covid-19 patients in your hospital at the peak of the crisis (non-cumulative)?

- 0-50
- 50-100
- 100-200
- 200 or more
- I don’t know

# F. Institution

What institution do you work in? This information will solely be used to assess the number of institutions that the information was derived from. It will not be published and it will be kept entirely confidential. Under no circumstance will it be shared with the institution.

- Institution (free text)
- I don’t want to share this information

# G. End of survey

Many thanks for your participation in the survey. If there’s anything else you’d like to share, please let us know in the text field below.

Please do not fill out this survey more than once. If you want to add information once you’ve submitted your answers, please contact ESNR at info@esnr.org.
